# New insights into the behavioral structure of Pikler educators: An application of T-pattern detection and analysis with THEME

**DOI:** 10.3389/fpsyg.2023.1081785

**Published:** 2023-02-27

**Authors:** Haizea Belza, Mariona Portell, Elena Herrán, M. Teresa Anguera

**Affiliations:** ^1^Department of Developmental Psychology and Education, University of the Basque Country UPV/EHU, Bilbao, Spain; ^2^Department of Psychobiology and Methodology of Health Sciences, Universitat Autònoma de Barcelona, Cerdanyola del Vallès, Spain; ^3^Faculty of Psychology, Institute of Neurosciences, University of Barcelona, Barcelona, Spain

**Keywords:** Pikler-Lóczy education, observational methodology, T-pattern, THEME, observation instrument, early childhood

## Abstract

**Introduction:**

The recent generalization of early childhood schooling has given rise to a need for the development of rigorous, specific training programs aimed at early education professionals (0–3 years of age). This work emanates from the unique nature of Pikler-Lóczy education as a reference for early childhood education centers, and its expertise in providing quality care in an everyday classroom situation. The aim of the study is to use T-pattern detection and analysis, within the framework of observational methodology, to identify relevant aspects of the *choreography* followed by Pikler educators during breakfast, and thus provide substantial material with a view to creating a training plan.

**Methods:**

Two expert educators in Pikler-Lóczy education were directly and systematically observed in their own classrooms, following a nomothetic, follow-up and multidimensional design. The observation instrument underwent a molarization process so that the detected elements would be more significant and understandable for novice educators.

**Results:**

Using THEME, the invariant aspects of the educators’ behavior were analyzed. The significance level for the critical interval was the THEME default (α = 0.005). The results show: on the one hand, changes expected in T-patterns in accordance with the observation instrument molarity; and on the other hand, stability in terms of the comparison between the two educators.

**Discussion:**

These results help us to identify the complex structure of the breakfast choreography, and how educators establish interaction with children. In addition to specific issues pertaining to Pikler-Lóczy education, the conclusions highlight the advantages of using T-pattern analysis within the framework of observational methodology, in order to dissect a mealtime routine in its natural context, and explain components of early childhood education intervention that satisfy basic childhood needs. This constitutes a starting point from which to generate instructive material for the training of professionals working in early education.

## 1. Introduction

Over the last few decades, early childhood schooling (0–3 years of age) has grown exponentially to become a generalized reality (European Commission, 2019). This has given rise to an increase in studies centered on the assessment of educational programs and interventions in nursery schools (Kaufman et al., 2015; Escolano-Pérez, 2020). A number of these studies warn of poor quality in such centers, especially in terms of educator-child interaction (Turnbull et al., 2009; Durlak, 2010; Hallam et al., 2016). This is largely due to the fact that this educational phase lacks its own identity (Herrán et al., 2014) for two reasons: on the one hand it has inherited educational traditions from successive cycles, introducing content that does not fit with the specific characteristics of early childhood (Sarmento and Ruiz, 2011); and, on the other hand the job is carried out in an intuitive, affectionate, maternal way, neglecting the professional dimension of daily tasks (Herrán, 2013; Falk, 2018a). Considering the proven effect of early experiences on human psychological development (Bick and Nelson, 2017), more and more authors are calling for a system rooted in the natural circumstances of infant daily life (Duncan and Magnuson, 2013; McLachlan et al., 2018), that helps to fulfill their early developmental needs (Csibra and Gergely, 2009; Bonawitz et al., 2011; Hallam et al., 2016; Tomasello, 2016; Salcuni et al., 2017). However, there are few rigorous training programs aimed at educators and early education professionals that provide a specific guide as to how to carry out everyday activities in the infant classroom, as well as how to assess progress in the performance of these activities.

Previous works have justified the interest and unique nature of the Pikler-Lóczy education (PikLEd) system as a reference for early education centers covering the 0–3 age group (Tardos, 2007; McCall et al., 2010; Herrán, 2013, 2018; Herrán et al., 2014; Salamon and Harrison, 2015). The PikLEd is an institutional childcare proposal exclusive to early childhood. It is based on over 70 years of practical and professional experience in the Lóczy Institute of Budapest (Pikler, 1968, 1971; David and Appell, 1986, 2010; Falk, 2002), which began as a foster home (1946–2011) but later became a nursery school (from 2006 to the present day). Assuming that an early experience of competence is crucial for the future shaping of the psyche (Bruner, 1972, 1973; Wallon, 1980; Pikler, 2018), this model revolves around two fundamental axes of everyday infant life: daily care and autonomous infant activity. It entails a set of original action protocols, known as *choreographies*, that transform everyday activities (for example, breakfast) into an educational field (Belza et al., 2019, 2020). The authors who have gone deeper into analyzing the practice of PikLEd have highlighted its relevance as a responsive care system (Hevesi, 2018; Pikler, 2018; Falk, 2018b) that is truly paedocentric (Falk, 2008; David and Appell, 2010; Herrán, 2013; Tardos and David, 2018; Vincze, 2018), and which facilitates infant socialization without violence (Kàllò, 2014, 2016; Pikler, 2018).

Over the last few years, a number of studies have provided new knowledge about PikLEd through the application of observational methodology (OM; Belasko et al., 2019, 2022; Belza et al., 2019, 2020; Sagastui et al., 2020, 2022). OM allows us to assess spontaneous behavior in its everyday context (Anguera, 1979, 2003; Portell et al., 2015). This methodological option offers valuable procedural resources for the study of daily life in the classroom and the adult-child relationships that are established therein (Anguera, 2001a). It also provides the objectivity and rigor essential for educational assessment, whilst at the same time affording the necessary flexibility for capturing the many, often complex processes of development and early learning in real life environments (Anguera, 2001b; Herrán, 2008; Early Head Start National Resource Center, 2013; Escolano-Pérez et al., 2017; Escolano-Pérez, 2020). OM offers guidelines for justifying and analyzing the quality of the records obtained using study dimensions and segmentation criteria of the spontaneous behavioral continuum in its natural context. Thus, OM involves the systematization of initial descriptive records – first dataset – through an observation instrument in order to obtain an equivalent code matrix – second dataset – that is built on the first (still qualitative data, where columns are dimensions/sub-dimensions, and rows are the successive units), which will be analyzed through specific quantitative techniques for categorical data. This way of connecting qualitative information and quantitative data has led to OM being considered as mixed-method in itself (Anguera and Hernández-Mendo, 2016; Anguera et al., 2017, 2018a; Onwuegbuzie and Johnson, 2021; Hitchcock and Onwuegbuzie, 2022).

Beneath the scope of OM, observational instruments were legitimized and applied, in order to capture details about the behavior of the expert PikLEd educator that facilitate the detection of two dimensional, instrumental and relational patterns in the daily performance of each everyday activity: breakfast, dressing and undressing, free play, etc (Belasko et al., 2019, 2022; Belza et al., 2019, 2020; Sagastui et al., 2020, 2022). This research shows that the habitual behavior of the expert PikLEd educator ensures the stability of the environment and the consistency of educational behaviors, these being understood as precise and adjusted ways of responding to the development of infant autonomy. Within the research line begun by Herrán (2013, 2014, 2016) and Herrán et al. (2014), important progress has been made in characterizing the action protocols or choreographies followed by these professionals in each educational field, thanks to the use of procedures designed to capture essential details of the sequence of educators’ instrumental actions and their connection with relational behaviors deployed with the child.

One of the everyday activities transformed into an educational field for PikLEd is breakfast. The systematic observation of this activity has revealed the sequence of basic actions that the educators repeat, as many times as necessary, so that the child has breakfast in a way that *suits them*, meaning that the child eats comfortably, calmly and quietly, in accordance with the hunger they feel at the time and their own perception of being full. These sequences are the *detours* (Wallon, 1985) they carry out in order to provide each child, at the table, with the utensils and food laid out on the countertop (Belza et al., 2019). The reiterative circularity of this instrumental sequence, together with the specific, unique and meaningful management of distances and postures (proxemic behavior), has shown that the expert PikLEd educator promotes the appearance of shared intentionality (Tomasello et al., 2005) in the development of mealtime routines (Belza et al., 2019). It is this shared intentionality that forms the basis of early cultural, instrumental and social learning (Tomasello, 2016). Furthermore, enlightening results have been obtained through analyzing the differentiated use of the utensils specific to the activity, along the lines of object pragmatics (Rodriguez and Moro, 1999) which views objects in terms of their social use and functions in everyday life, functions which children gradually appropriate thanks to adult-instigated mediation. Significant relationships were detected between various breakfast utensils with differing complexity and each child protagonist, showing that the educators adapt, and provide utensils depending on the level of autonomy and interest of each child (Belza et al., 2020).

The evidence provided by the aforementioned studies about the breakfast activity was made possible thanks to the diachronic analysis of observational data (Anguera et al., 2021); more specifically, lag sequential analysis (Bakeman and Gottman, 1989; Bakeman and Quera, 2011; Quera, 2018) and polar coordinate analysis (Sackett, 1980; Anguera, 1997). Lag sequential analysis detects regularities in behavior, making patterns of behavior visible, whereas polar coordinate analysis enables the vectorization of behavior, creating a complete map of interrelationships between behaviors. These techniques enabled the characterization of significant parts of the choreography, but not the choreography as a whole. A third very important technique for diachronic analysis in OM is based on the algorithm developed by Magnusson (1996, 2000) and used in THEME software, which makes the description and detection of complex real-time patterns possible. Its basic concept is the T-pattern, a statistical, hierarchical, self-similar (pseudo-fractal) pattern, recurring with significant translation symmetry since detected in human, animal, and brain network behavior (Casarrubea et al., 2015, 2018; Magnusson, 2020). T-pattern detection and analysis (TPA) with THEME has led to new insight into the structure of behavior. This third technique used for diachronic analysis in OM may be the best option for a complete characterization of the choreography. Until now, the use of TPA within the framework of OM, in the field of education, has made it possible: to identify patterns of academic persistency in pre-school age-groups (Santoyo et al., 2020), to examine the development of cognitive skills in infancy (Escolano-Pérez et al., 2019; Escolano-Pérez, 2020), to evaluate teaching-learning strategies in motor skills (Castañer et al., 2009), to assess the interaction and communication models of physical education teachers (García-Fariña et al., 2018; Camerino et al., 2019; Valero-Valenzuela et al., 2020), and to design and assess training programs focused on the professionals’ change in behavior (Portell et al., 2019; Sene-Mir et al., 2020).

Therefore, through the use of TPA within the framework of OM, it is possible to facilitate the design and evaluation of formative interventions of PikLEd aimed at novice educators; and this application would allow us to compensate for an important deficiency in the generalization of the PikLEd model. The Lóczy Institute in Budapest – now the Emmi Pikler nursery school – rigorously prepares its educators *in situ,* with periodical observations of their actions with the children which are then discussed by the pedagogic team as a whole (Kelemen, 2016). However, in other centers there is no training material for novice educators associated to evaluation guidelines, that would allow professionals to track the acquisition process of essential elements of the choreography (what to do) and non-verbal communication (how to do) inherent to PikLEd. The general aim of this research is to provide new insight into the choreography followed by the expert PikLEd educator, which is considered seminal for the creation of an innovative training plan that includes T-patterns as elements of a self-modeling strategy (Dowrick, 1999, 2012). Based on the main dimensions that have been established to structure the breakfast activity (Belza et al., 2019), the specific aims are: (1) to adapt the instrument *Giving Breakfast in the Emmi Pikler Nursery School, GBEPNS* (Belza, 2020) which increases the molarity of its instrumental and proxemic dimensions, in such a way as to make it easier for an educator who is not an expert in PikLEd to understand the training material created from its elements; (2) to identify invariant aspects that structure the expert educator’s behavior by analyzing the T-patterns from both an intra-individual perspective – between patterns obtained from the same expert educator when modifying the molarity of the observation system –, and an inter-individual perspective – between the behavior patterns of different expert PikLEd educators.

## 2. Materials and methods

This work forms part of a line of research into PikLEd developed under the scope of OM. In accordance with the design classification established in OM (Anguera et al., 2001), the study design as a whole is nomothetic, follow-up and multidimensional (N/F/M design), since the observation units are greater than one; there are two educators, to be exact. However, it is worth qualifying that, for each educator, the design used is ideographic given that the behavior of each one was observed and recorded as an independent unit, with an intra-session and inter-session follow-up over a 3 month period; and is multidimensional since it includes different criteria in the observation instruments used to record and code behavior (I/F/M design). This study applied systematic, direct, non-participative observation.

### 2.1. Participants

Eligibility criteria were established for the educators so as to increase the probability of the behavioral records obtained being representative of the PikLEd model. These criteria are: (1) having done their professional training in the Lóczy foster home itself, under the direction of the pediatrician Emmi Pikler; and (2) having actively taken part in the continuous training programs organized by the institution. Furthermore, applying the logic of multiple case study repetition (Yin, 2014), this study seeks to integrate most of the school educators involved in the breakfast activity.

There are three classrooms in the Emmi Pikler nursery school in Budapest, with a maximum of 12 children and three educators in each one. Applying the aforementioned criteria, two educators were selected (henceforth, Educator-1 and Educator-2) who habitually manage breakfast in their groups. Various studies within the OM framework show that, despite working with a reduced number of participants, the intensive monitoring of their everyday activity makes it possible to collect a large quantity of data and extract insightful conclusions about the studied reality (Rodríguez-Medina et al., 2018; Belza, 2020; Lapresa et al., 2020; Sagastui et al., 2020; Arias-Pujol et al., 2022).

In terms of the groups, it is worth highlighting that this school endeavors to maintain the same three educators in each group in order to establish a close relationship with the children as well as to enable a continuous monitoring of the children. For this reason, the placing of children into groups is not done in accordance with the age criterion, but depending on the number of available places in each classroom and the characteristics of the children themselves. To be specific, Educator-1’s group was made up of four boys and eight girls between the ages of 26 and 47 months at the start of the observations. Educator-2’s group was made up of five girls and five boys aged between 20 and 36 months. As breakfast is an option offered to all the children, participation varied from session to session.

It was agreed with the school directors to film one session per week in each classroom first thing in the morning, from 8.30 to 9.30, over a 3 month period. As recordings are frequently made in the classrooms, both the educators and the children are familiar with the presence of a camera. This is undoubtedly a relevant aspect to consider in relation to reactivity (Behar and Riba, 1993).

A total of 28 sessions were filmed over 3 months, 11 in Educator-1’s classroom and 17 in that of Educator-2. The acceptance criteria for the sessions was established in line with the following aspects: (1) Observability of the educators and their activity: the camera follows them continuously from the moment the first child arrives to have breakfast until the last breakfast is cleared away; (2) Inter-session consistency (or fulfillment of formal minimums of situation and space homogeneity) – all the sessions correspond to the same two classrooms in which the activity takes place, mainly in the dining room area or in the adjoining lobby or bathroom; (3) Intra-session consistency (or temporal completeness of the criteria that define the session) – all the sessions must temporally cover the aim, which is to serve and complete breakfast within the established time slot. The following exclusion criteria were formulated: (1) Change of classroom for organizational or maintenance reasons; (2) Insufficient recording time to analyze the complete breakfast of at least one child. Despite 25 sessions being recorded, the application of this last criterion excluded three of Educator-2ʼs sessions from the analysis. Finally, the sample of analyzed breakfasts was made up of 10 sessions in Educator-1’s classroom and 12 sessions in that of Educator-2. Each session lasted between 10 and 40 min, depending on how many children had breakfast and what time each child began.

The research project was assessed and approved by the Committee for Ethics in Research and Teaching from the University of two of the authors (INA0139), after obtaining the informed consent of the two educators and gaining the permission of the authorities responsible for the Emmi Pikler nursery school in Budapest, in accordance with Law 41/2002 of patient autonomy. The data has been treated in line with the regulation included in RD1720/2007 of development of the Organic Law 15/1999 of Personal Data Protection and in Law 14/2007 of Biomedical Research.

### 2.2. Instruments

#### 2.2.1. Observation instrument

The observation instrument used is an adaptation of the field format *Giving Breakfast in the Emmi Pikler Nursery School, GBEPNS* (Belza, 2015, 2020; Belza et al., 2019, 2020), an *ad hoc* creation (Anguera et al., 2007) to observe the habitual behavior of the expert PikLEd educators during breakfast. This instrument is made up of six dimensions grouped into two macro-dimensions: instrumental, associated with the material conditions of the specific activity of breakfast; and relational, appertaining to the interaction and communication that the educators establish with the children at different levels (paralinguistic, proxemic, kinesic, and verbal). Each dimension is displayed in a feature list type catalog of behaviors (Anguera et al., 2018b).

GBEPNS was developed for a very molecular level of description, which allows us to collect the smallest details of the educators’ behavior during breakfast, but which may hinder the subsequent explanation and interpretation of this behavior when it is dissected. In accordance with the aims of this study, the initial field format underwent a molarization process through which the catalogs of behaviors, spaces and objects (utensils in this case) were redefined and reordered, with a view to making the detected elements more significant and understandable for novice educators. In order to carry out this adaptation of GBEPNS, in its instrumental and proxemic dimensions (henceforth GBEPNS-IP), a dynamic process of initial formulation of tentative groups was followed, subsequently modified in line with an empirical-inductive strategy, and once again, consistent with a deductive strategy. A group of PikLEd and OM experts intervened in this process. Similar aspects were grouped together in relation to their educational significance and their transferability to other classrooms, maintaining the specifics in terms of behavior related to children. The result of this process of adaptation of the GBEPNS-IP instrument is referred to as GBEPNS-IPv2. Table 1 shows the structure of GBEPNS-IPv2 and its correspondence with GBEPNS-IP.

**Table 1:** Dimensions, sub-dimensions, and catalogs of the behaviors/spaces/utensils of the GBEPNS-IPv2 instrument and their concordance with the catalogs of the GBEPNS-IP instrument.

GBEPNS-IPv2			GBEPNS-IP
Dimensions	Sub-dimensions	Catalogs	Catalogs
Instrumental	Space	Counter	Counter 1, counter 2, counter 3, breakfast basket, trashcan, paper tissue repository, wall closet
		Table	Tables 1, 2
		Her place/chair	
		Other sub-area of the dining room	Door, entrance, corridor, handrail
		Other area of the classroom	Play area 1, play area 2, sanitation area, washbasin, lobby, lobby window
	Action	Greets/welcomes	Greets from where she is, welcomes approaching the door, farewell to parents ritual
		Offers food	
		Puts	Replaces
		Takes/leaves/goes in search	Moves
		Serves	
		Other breakfast action	Prepares food,/spreads/cuts/opens, Puts away, Organizes, throws away leftovers, cleans objects, sweeps, mops
		Gets child to use napkin	
		Collects childʼs breakfast	Takes it away if uneaten
		No action	
		Other (hers)	Puts glasses on/blows nose/washes hands, writes in notebook, opens-closes handrail
	Utensil	Glass	
		Jug/bag/ recipient	
		Glass and jug	
		Bowl	
		Plate	
		Napkin	Handkerchief (nose)
		Breakfast kit: various	
		Other (hers)	Knife,/scissors, fork, spoon, mop, broom, cloth, stool, notebook and pen, other
		Soother/transitory object	
		None	
Proxemic behavior	Static alone	On foot	
		Bent forward	Leaning forward
		Crouching/kneeling	Kneeling, sitting on knees
		Sitting	
	Static with child	None	
		Takes/places him/her	Takes him/her in her arms, places him/her on her lap, places him/her between her legs, Puts her arm around him/her, lowers him/her to the floor
		Surrounds him/her in order to maneuver	
		Next to her	
		Opposite	
	Movement	None	
		Approaches	
		Moves away	
		With child	Follows him/her, carries him/her in her arms, they go hand in hand, they walk in parallel, the child follows her
		Without relationship to the child	

It can be noticed that each dimension is broken down into three sub-dimensions so as to structure the elements and guarantee mutual exclusiveness. The instrumental dimension includes the spaces in which the activity takes place, the educator’s actions (what she does) and which utensils she uses. Proxemic behavior refers to how the educator uses the space, and covers two facets: the static character that refers to the choice of place and posture within a space; and the dynamic character that involves the set of movements inherent to the activity itself. In aiming to capture the interactive dynamic that the educator establishes with the children in her care, the static facet is divided into two criteria: that of solitary static which refers to posture or position; and that of static in relation to the child present with whom she can make contact or stay very close to without moving. Once the catalogs of behaviors, spaces and utensils for each sub-dimension had been compiled, a system of alphanumerical codes was created to enable the coding of each of these elements.

#### 2.2.2. Recording and analysis software

The free software program HOISAN, version 1.6.3^1^ (Hernández-Mendo et al., 2012, 2014) was used to record and code all the sessions based on the system established in the field format. This program enables the databases to be exported to MS-EXCEL. Thus, the record files can easily be transformed into the syntactic structure of other programs for data analysis. In this case, we used the program THEME 6 Edu (Magnusson, 2000) for data management and analysis, which is freely available for download.^2^

### 2.3. Procedure

From the observation instruments GBEPNS-IP and GBEPNS-IPv2 we obtained records of the 22 sessions. The basic unit in field format record is the configuration or co-occurrence of codes from different dimensions. The configuration is formed by as many codes – at most – as there are number of sub-dimensions. When a behavioral aspect changes, the next configuration begins, thus enabling an exhaustive record of behavioral flow. In this way, the maximum systematization of the record is achieved, which produces large code matrices. At the same time, the duration that includes information about both the position of the code within the sequence and its occurrence, was used as a primary record parameter (Anguera et al., 2001; Bakeman and Quera, 2011). This enables the detection and analysis of T-patterns (TPA).

The HOISAN computer program allows us to obtain and store a large quantity of data and subject them to quality control. The files with the record made in HOISAN were exported to MS-EXCEL to be prepared in accordance with the instrument GBEPNS-IPv2. In this way two new code matrices were obtained, one for each educator, which were transformed into the syntactic structure compatible with the THEME program. This program was used to analyze the data of Educator-1, obtained with GBEPNS-IP and with GBEPNS-IPv2; and those of Educator-2, obtained with GBEPNS-IPv2.

### 2.4. Data quality check

Both qualitative and quantitative quality control procedures were carried out on the data. Consensual concordance (Anguera, 1990) took place in the last stage of construction of the observation instrument. This procedure, that seeks to achieve convergence between two observers, is increasingly present in OM (Arana et al., 2016). To this end, two of the authors of this study jointly carried out record trials with 15% of the sample, to decide which code to assign to each of the observed behaviors. The definitions of the behaviors in the catalog were therefore shaped and nuanced, thus strengthening the observation instrument.

In terms of quantitative quality control of the data, Krippendorff (2013) alpha coefficient was calculated, both intra-observer and inter-observer, *via* the HOISAN program. Ten per cent of the sample was recorded twice at different times for comparison, and once more by an external observer. In both cases the coefficient value was satisfactory since the degree of agreement between the two observers was 0.89, whilst it was 0.92 between the two records at different times.

### 2.5. Data analysis

According to Magnusson (2016), the THEME software considers data as a series of points that represent the occurrence of events during a period of observation (in TPA the event refers to the configuration or chain linking of codes from each sub-dimension of the field format in each of the event-types). The number of appearances of an event-type, divided by the duration of the observation period, produces the mean probability of this event-type occurring in a given unit of time, which is later used as the reference probability for the detection algorithms. When THEME detects an occurrence of a followed by B within a critical interval, it generates a simple T-pattern (AB). The critical interval is a key concept in TPA, and it refers a particular relationship between pairs of events in a time series—for an extended explanation of this concept see Magnusson (2000) and Casarrubea et al. (2015, 2018). The occurrences of simple T-patterns become events that subsequently constitute focal event-types in the next detection level. This process is repeated upwards level by level, in search of critical interval relationships with the patterns detected in previous levels, in order to generate ever more complex T-patterns.

This study independently analyzed the three aforementioned databases – in each one, the included sessions were concatenated into one multi-sample base – using the same search and selection criteria parameters to compare the obtained T-patterns (Amatria et al., 2017; Pic et al., 2021; Pic and Jonsson, 2021). More specifically, the search parameters applied were (Magnusson, 2017): (1) an occurrence frequency greater than 3; (2) a significance level of 0.005 (0.5% probability that the critical interval was randomly produced); (3) a minimum pattern occurrence in 50% of the sample. TPA detection was validated by simulation, through data randomization with shuffled and rotated randomized versions of the data sets. We used the standard number of randomization-searches (5 runs per type). The only accepted patterns were those for which the concordance probability between the randomized data and the real data was zero.

Given that the aim of this study was to analyze individual patterns, it is necessary to operationalize the selection conditions to make them comparable. Since the aim is that the patterns help to characterize the structure of everyday educational behavior as a whole, three selection criteria were applied to the patterns detected by THEME: (1) maximum length (patterns that include the greatest quantity of event-type or configurations); (2) maximum level of hierarchical structuring; (3) greatest session time coverage.

## 3. Results

In relation to the behavior of Educator-1, 4,716 configurations or data points were analyzed, whilst 2,319 configurations were analyzed in the case of Educator-2. The number of configurations was maintained both with GBEPNS-IP, and with GBEPNS-IPv2. In line with the second specific aim, we present the results below, in two sub-sections that show the intra-individual and inter-individual comparisons between patterns.

### 3.1. Changes in the structure of Educator-1ʼs behavior with the GBEPNS-IP and GBEPNS-IPv2 instrument

**Figure 1:**
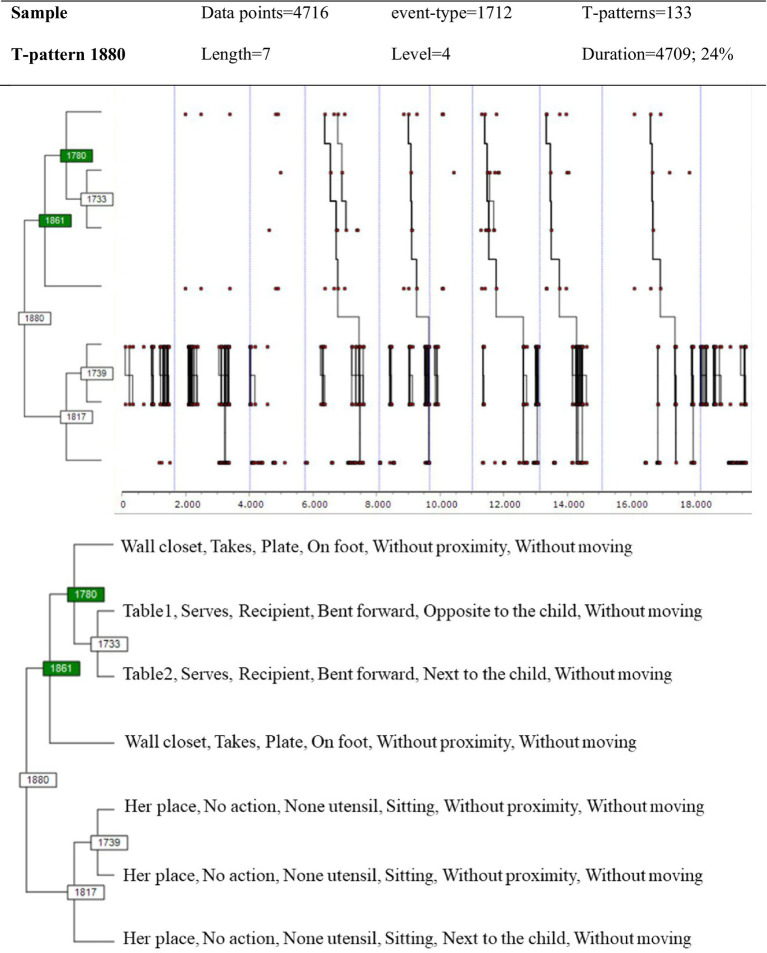
T-pattern of Educator-1ʼs behavior obtained with GBEPNS-IP.

Starting from the code matrix based on GBEPNS-IP and applying the parameters and criteria defined in the data analysis section, the obtained pattern, among the 133 detected, is shown in Figure 1. It can be seen that in this T-pattern there is a certain structuring of Educator-1ʼs behavior, made up of two sub-sets of simpler patterns: on the one hand, the pattern shows the hidden structure concerning the management of breakfast in the different sessions (*takes an item from the cupboard*, *serves table 1*, *serves table 2, returns to the counter*); and on the other hand, a highly recurrent pattern that reflects accompaniment when there is *no action*.

**Figure 2:**
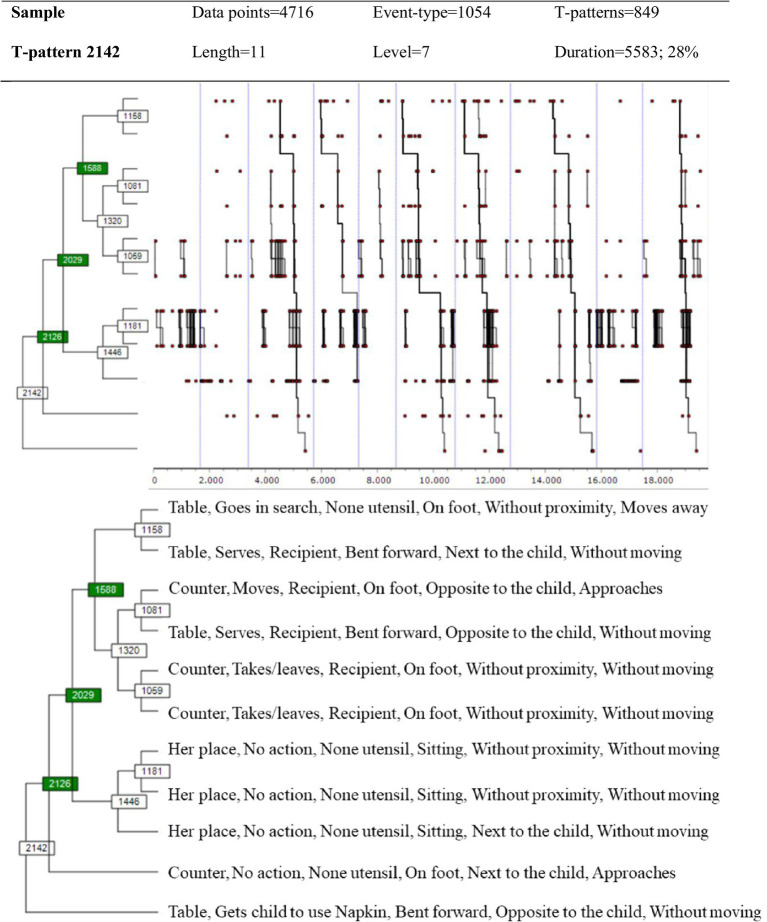
T-pattern of Educator-1ʼs behavior obtained with GBEPNS-IPv2.

Figure 2 shows the pattern resulting from the application of the selection criteria for the 849 T-patterns detected with the same search parameters, when using GBEPNS-IPv2. The level of behavioral structuring in this case is visibly greater, above all in reference to the instrumental actions; and at the same time, it integrates the simple pattern *no action*, again demonstrating the high recurrence of this event. Furthermore, this complex T-pattern reveals the appearance of new educational behaviors, such as closing the activity with the *gets child to use the napkin* action.

### 3.2. Stability in the behavioral structure between Educator-1 and Educator-2 with the GBEPNS-IPv2 instrument

**Figure 3:**
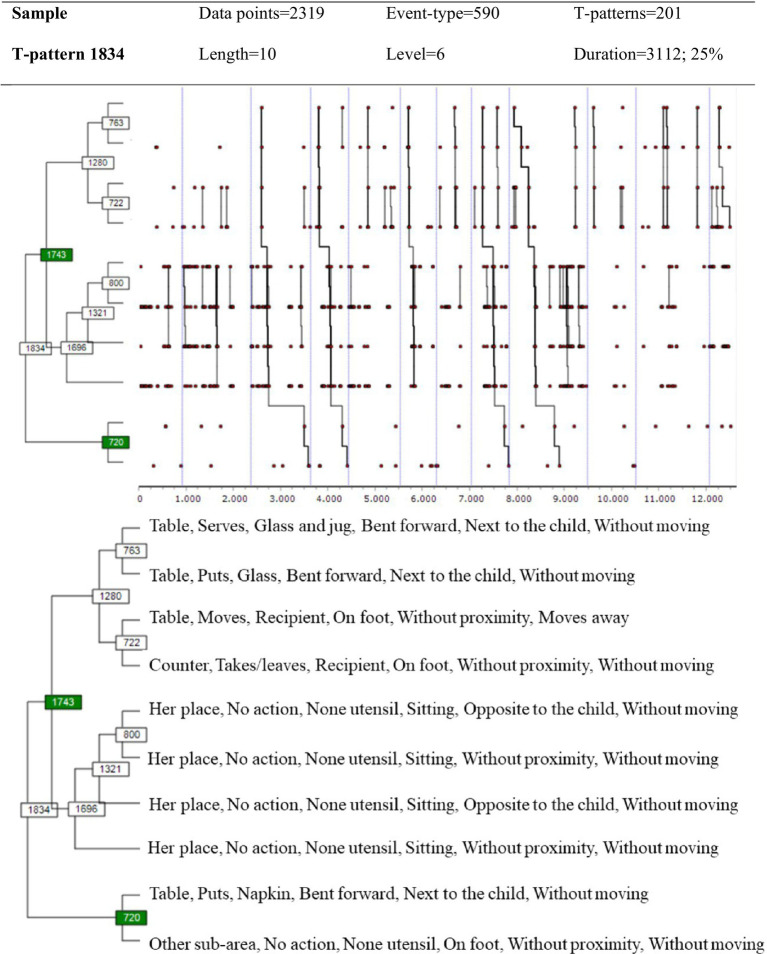
T-pattern Educator-2ʼs behavior obtained with GBEPNS-IPv2.

Using the same selection criteria as in the previous analysis, we obtained the pattern shown in Figure 3 of the 201 T-patterns detected in the behavior of Educator-2. In this pattern there are three event-type sub-sets, which, although differing slightly in how they are structured in relation to Educator-1ʼs T-pattern, they practically replicate its composition: chain linking of instrumental actions (*serves, puts, moves, takes/leaves*) that alternate movements (*moves nearer, moves away*) between the *counter* and the *table*; again, there are repetitions of the central sub-set of accompaniment when there is *no action*, and finally, the use of the *napkin*.

## 4. Discussion

This study focused on two dimensions: one of instrumental behavior, and the other of the expert PikLEd educator’s non-verbal behavior during the breakfast activity. The relevance of both these dimensions, instrumental and proxemic, has been revealed in previous studies as backbones of behavior flow on two levels – physical/instrumental and human/relational (Belza et al., 2019; Belza, 2020). Furthermore, due to their high degree of perceptibility and relationship with reality, these dimensions offer better training opportunities for novice educators than others perceived in the GBEPNS instrument. It was expected that the detection of T-patterns would identify greater levels of structuring in everyday behavior compared to other diachronic analysis techniques, and thus provide an account of the choreography as a whole. Below we discuss the results obtained relating to Educator-1ʼs behavior, in order to compare the identifying and explanatory capacity of the two observation instruments, GBEPNS-IP and GBEPNS-IPv2. Subsequently, we explain the T-patterns of Educator-1 and Educator-2, obtained with GBEPNS-IPv2, in order to clarify the potential behavioral homogeneity between the two.

Firstly, we can see that the variability of event-types drops to almost half when GBEPNS-IPv2 is applied to the analysis of Educator-1ʼs behavior, compared with the use of GBEPNS-IP. This fact adds more consistency to the record and reduces the dismembering of the behavior by residual aspects, without prejudicing significant information, given that the versatility of the events compared to the total is still high. The amount of different event-types, interpreted as behavior variability, shows that the PikLEd expert constantly adjusts to each moment, circumstance and child (Kàllò, 2016; Hevesi, 2018). If one of the defining aspects of early education is the educators’ capacity to adapt their practice to the children’s basic needs (Durlak, 2010; Escolano-Pérez, 2020), then PikLEd prioritizes the establishment of a close, continued relationship between the educator and the children in her/his care. In this way, everyday interactions can be furnished with the particular and individualized traits that come from mutual knowledge and which are so necessary at early ages (Falk, 2008). Despite this variability, the obtained results relating to Educator-1 support the use of the GBEPNS-IPv2 instrument which, combined with TPA, has allowed us to discover a more complex everyday behavioral structure for the expert PikLEd educator. It also provides new aspects of the choreography during breakfast whilst integrating the findings of previous studies (Belza et al., 2019, 2020) – the basic sequences that instrumentally order this mealtime routine and its cultural-socializing character. Therefore, the results sustain the idea that THEME is effective in obtaining clarifying patterns of this intervention in its natural context – a nursery school classroom.

Secondly, the aim was to check whether applying the most molar GBEPNS-IPv2 instrument and the same analysis strategy with THEME would lead to Educator-1ʼs results being replicated in Educator-2. Before going on to compare the patterns obtained from both, we want to highlight differences in the two breakfast situations. Since the temporality of the sessions was marked by the children’s attendance, together with the desire and rhythm in the breakfast itself, the duration of the sessions was disparate. This means, for example, that Educator-2ʼs sample, although including a greater number of sessions than that of Educator-1, produced a lower quantity of data due to fewer children having breakfast and the sessions being shorter. However, this fact does not appear to impede the detection of a similar structure in the behavior of both educators. The two T-patterns obtained are made up of event-type symmetries and have similar characteristics: (1) Chain linking of instrumental actions for breakfast management, (2) Recurrent cycles of accompaniment without action, (3) Finalization using a napkin. These results provide evidence about the stability of everyday intervention at breakfast time in this school, in two senses: On the one hand, they add evidence about the detours (Belza et al., 2019) made by the educators from the counter where the utensils – that they move, put down or serve, moving closer to the children at the table – are kept, until they return to the counter. Furthermore, the T-patterns obtained illustrate that it is the proxemic dimension that characterizes this instrumental sequence; guaranteeing coherence between the physical and human elements that make up the early educational breakfast environment, and showing the relevance of the intentional use of the space when it comes to establishing educator-child relationships. The expert PikLEd educator’s choice of posture and distance is neither accidental nor improvised, but is a response to the level of intervention/interaction that she needs to establish for the *joint action* (Tomasello et al., 2005; Sagastui et al., 2022). On the other hand, that both educators should display such similar behavior is due to the fact that, since its beginnings as a foster home, the main aim of the Lóczy Institute has been to provide the emotional security necessary for the healthy psychological development of institutionalized infancy (Pikler, 1971). Such important acquisitions in the earliest years cannot be integrated into the personality being structured if the child does not carry out her/his experiences within the framework of an interpersonal system of stable, continuous relationships (Falk, 2008). To this end, the aspects that make up each care were detailed and examined, in order to organize them in the form of a choreography, and thus ensure the continuity and homogeneity in the daily tasks of the educators who interact with the children (Herrán, 2013).

On an educational level, two other components of this choreography stand out. The *accompaniment without action* pattern is where the educator sits on her chair, and from that position accompanies both the breakfast of the child sitting at the table next to, or opposite her, along with the rest of those present, including those who are already in the play area. This sub-set of event-types is a recurring loop that is inserted into the choreography. They are attention intervals dependent on the aspiring action of the children, which allows the educator to attend and respond to the requirements of each moment in an appropriate way, even preempting them, thus displaying responsive care (Hevesi, 2018; Pikler, 2018). Her presence increases as the session progresses; something which is logical given that most of the children have been served, are finishing or are already playing. Even so, the fact that this pattern is repeated throughout the session characterizes the delimited space known as “her place” – i.e., her chair, situated near to the breakfast table, between this and the counter – as a transitional space between one action and the next, or between one interaction and the next. This shows another way of organizing and regulating the activity through the use of the space. Finally, it is worth highlighting the appearance of a previously unknown action – the culmination of the breakfast, consisting in the use and placement of the napkin, which closes the activity. In fact, in the case of Educator-2 this action takes place in another sub-area of the dining room – the handrail or corridor that connects to the play area. Therefore, the napkin, which denotes a certain degree of acceptance of social norms or conventions by the child (Belza et al., 2020), marks another important transition: from the breakfast activity to the one that immediately follows, which is play. Once the basic, affective needs have been covered – dietary, emotional and adaptable to nursery school – the children can then unleash their abilities associated with autonomous activity and free play. According to Tardos (2014), attending school involves facing certain daily challenges on the children’s part. In this sense, breakfast constitutes an activity that supports the transition from home to school; each child receives a welcome and a personalized breakfast so that they feel expected and comfortable (Tardos, 2014; Belza, 2015). These transitions are fundamental in infant daily life in order to ensure security and welfare. The expert PikLEd educator prepares them in such a way as to create a solid framework in which children can integrate references that help them settle, find their place and build structure (Falk, 2008).

In short, what can be deduced from this work, in addition to the specific matters relating to PikLEd, are the advantages of using TPA within the OM framework to dissect an everyday activity in its natural context, and explain the components of early education intervention that satisfy basic infant needs. The results reveal a complex structure of the everyday behavior of these educators, which provides insightful information about the breakfast choreography, and how the expert PikLEd educators establish interaction with the children. This represents a starting point from which to generate instructive material for training early education professionals. In this sense, since the detected patterns integrate and structure various dimensions of instrumental and non-verbal behavior, they allow us not only to assess the result of such training, but also to create feedback during the process itself (Santoyo et al., 2020; Sene-Mir et al., 2020). Therefore, this work makes a significant contribution in terms of addressing the gap that exists in specific training programs for professionals working with the 0–3 age group; a gap that has been highlighted by various authors (Herrán et al., 2014; Escolano-Pérez, 2020).

As previously mentioned, one of the limitations of this study was the sample composition. Since the emphasis was on carrying out a detailed study of everyday interactions, the number of sessions was somewhat limited, in addition to its heterogeneity for enabling the analysis of the relationships and integration of the different dimensions of the educators’ behavior with each child in particular. To be exact, it would be interesting to be able to integrate the role of kinesic behavior into these patterns, so that instruction in responsive communicative abilities in early education could be added to the training plan.

## Data availability statement

The raw data supporting the conclusions of this article will be made available by the authors, without undue reservation.

## Ethics statement

The studies involving human participants were reviewed and approved by the Committee for Ethics in Research and Teaching from the University of the Basque Country (UPV/EHU). Written informed consent to participate in this study was provided by the participants’ legal guardian/next of kin. Written informed consent was obtained from the individual(s) for the publication of any potentially identifiable images or data included in this article.

## Author contributions

All authors listed have made a substantial, direct, and intellectual contribution to the work and approved it for publication.

## Conflict of interest

The authors declare that the research was conducted in the absence of any commercial or financial relationships that could be construed as a potential conflict of interest.

## Publisher’s note

All claims expressed in this article are solely those of the authors and do not necessarily represent those of their affiliated organizations, or those of the publisher, the editors and the reviewers. Any product that may be evaluated in this article, or claim that may be made by its manufacturer, is not guaranteed or endorsed by the publisher.

## Footnotes

^1^ www.menpas.com

^2^ www.patternvision.com
